# Experience from a Multi-Disciplinary Team against COVID-19: A Healthcare Perspective

**DOI:** 10.3390/ijerph18041678

**Published:** 2021-02-09

**Authors:** Alvin Cong Wei Ong, Clarice Li-Phing Wee, Wei Lin Lee, Lee Gan Goh, Ghee Hian Lim

**Affiliations:** 1 Department of Post-Acute and Continuing Care, Jurong Community Hospital, National University Health System, Singapore 609606, Singapore; 2 Ng Teng Fong General Hospital, National University Health System, Singapore 609606, Singapore; Clarice_Wee@nuhs.edu.sg (C.L.-P.W.); Wei_Lin_lee@nuhs.edu.sg (W.L.L.); Ghee_Hian_Lim@nuhs.edu.sg (G.H.L.); 3 Department of Family Medicine, National University Health System, Singapore 119228, Singapore; mdcgohlg@nus.edu.sg

**Keywords:** coronavirus disease 2019 (COVID-19), severe acute respiratory syndrome coronavirus 2 (SARS-CoV-2), community care facilities, isolation facilities, primary care, pandemic medicine

## Abstract

Globally, the capacity of healthcare systems across continents has been strained and put to the test with the emergence of the Coronavirus disease 2019 (COVID-19) pandemic. The timely need to ensure the availability of healthcare facilities to isolate and manage the surge in COVID-19 cases without overwhelming existing hospital capacity has posed challenges in many countries. In this paper, we discuss the conceptualisation, preparations and operationalisation of a community healthcare facility that was set up within a short time frame to attend to the convalescent needs of a large number of COVID-19 patients in the early phase of handling the pandemic. In the first month of operations, we monitored a total of 2129 clinical encounters, with the majority of patients between 17–35 years of age and between day 2 to day 6 of illness upon admission. Overall, there was a good outcome for the patients, with only 2.3% requiring transfer back to restructured hospitals. There was also no mortality. We hope that the sharing of our experiences of the challenges and learning lessons gleaned may be useful to guide individuals in planning for the future preparedness of healthcare systems in managing pandemics.

## 1. Introduction

### 1.1. Background

On 11 March 2020, the World Health Organization declared Coronavirus disease 2019 (COVID-19) to be a global pandemic [1]. COVID-19 is caused by severe acute respiratory syndrome coronavirus 2 (SARS-CoV-2). The first known case was reported in Wuhan, China, in December 2019 and the disease rapidly spread globally and was beyond the four million mark by 9 May 2020 [2]. In Singapore, the first case definition was established on 2 Jan 2020 and the first case of COVID-19 was diagnosed on 23 Jan 2020. Between 30 March 2020 and 4 April 2020, several COVID-19 clusters were identified at foreign worker dormitories [3]. On 5 April 2020, dormitories began to be designated as isolation areas [3,4]. As the number of COVID-19 cases increased exponentially with the outbreaks in dormitories housing foreign workers, Singapore shifted strategy in the care of the COVID-19 patients by treating majority in community facilities instead of hospitals. This freed up precious and expensive hospital resources [1]. In April 2020, three community isolation facilities were set up in Singapore, namely in D’Resort NTUC Chalet, Singapore Expo and Changi Exhibition Centre [5]. These facilities were built to isolate and care for patients who were asymptomatic or had relatively mild symptoms. This strategy of converting public facilities such as exhibition halls into temporary hospitals was also practised in other countries [6].

#### A Familiar Global Strategy

The idea of repurposing existing public venues such as exhibition centres and stadiums into large-scale temporary healthcare facilities or hospitals was used to tackle the shortage of beds in accommodating the huge demands of the pandemic. One of the very first such facilities was Fangcang shelter hospital in Wuhan, the very first city in China to report the outbreak [7,8]. It was regarded as purposeful and novel as such facilities could be built in a relatively short time due to the relative ease in retrofitting the existing infrastructure and the large numbers of patients who could be isolated and housed within. These temporary hospitals emerged as one of the most efficacious methods to limit the transmission of the virus and to reduce mortality. This rapidly expanded the capacity of and empowered the local healthcare system to deal with the pandemic [8].

In South Korea, which experienced a dramatic rapid surge of COVID-19 infections within the first six weeks of the initial outbreak in January 2020, the overwhelmed hospitals compelled a sizeable number of confirmed cases to stay at home instead of being cared for in a dedicated healthcare facility. The report of an out-of-hospital death on 27 February 2020 involving a confirmed case of COVID-19 who deteriorated and passed on while waiting hospital admission due to inadequate hospital resources was indeed alarming [9]. Community treatment centres, which shared a similar model, function and purpose as temporary hospitals, were set up. These facilities were designed to care for confirmed cases with mild symptoms––not those in need of advanced medical care––requiring isolation and active surveillance for any deterioration in condition. These models were replicated in other countries such as the United States, where they were termed “alternate hospital sites” (AHSs), and were used in locations such as Rhode Island to supplement the hospital capacity [10].

### 1.2. Community Care Facility (CCF)/Community Recovery Facility (CRF) @ Big Box

Big Box is an eight-storey warehouse mall located in western Singapore. It is situated adjacent to Ng Teng Fong General Hospital (NTFGH) and Jurong Community Hospital (JCH), which are part of the National University Health System (NUHS), one of the three major public healthcare clusters in Singapore. NTFGH and JCH are collectively referred to as JurongHealth Campus (JHC). It was earmarked to be deployed as a CCF/CRF facility to be managed under NUHS by the Ministry of Health of Singapore. The CCF/CRF@Big Box aimed to provide essential primary healthcare services to a generally stable population with the aim of identifying early signs of complications arising from COVID-19 infection.

## 2. Materials and Methods

### 2.1. Aim

The aim of this paper is to share and describe the conceptualisation, preparations, operationalisation and key findings of a community healthcare facility that was set up within a short time frame to attend to the convalescent needs of a large number of COVID-19 patients in the early phase of handling the pandemic.

### 2.2. Concept of Operations

The CCF was set up to operate as a holding facility for young COVID-19 patients with mild or no symptoms to allow time for them to recuperate and tie them over for the period of time when they may be infectious. They were thus expected to be able to perform self-monitoring for their health status (such as performing basic vital signs measurements). These patients required isolation and were unable to isolate in their usual dwellings (e.g., dormitories).

### 2.3. Key Considerations

#### 2.3.1. Organisational Structure

A clear command and control hierarchical structure was paramount to ensure proper assumption, delegation and execution of the key responsibilities of each member. Each key lead was familiar with the scope of his or her assigned roles and adhered to the structured reporting regime. The operational wing of the CCF worked closely in tandem with the main command centre to ensure the clockwork reliability of the ground operations as well as to facilitate the malleability of the team to handle contingencies and ever-evolving changes in protocols, which were updated on a regular basis in consultation with the authorities and epidemiological climate.

#### 2.3.2. Communications

An effective and barrier-free communication that allowed timely dialogue between the command centre and ground team was essential for prompt dissemination of crucial information as well as changes in workflows. This was applicable not only to the conveyance of information from the leads to the ground staff; the conveyance of feedback and operational issues from the CCF team back to the command team was likewise important for well-timed interventions to be made known and initiated by the decision-wielding management in resolving the potential obstacles and concerns on the ground. 

Daily end-of-the-day debriefs known as “post-action reviews” were conducted by representatives from the command team, CCF team leads, managing agents and the ground staff. These sessions served as daily huddle sessions to facilitate information sharing between both parties as well as discussion platforms to troubleshoot issues and rectify identified obstacles. Issues that remained unresolved at the end of the daily sessions were promptly escalated to higher authorities and decision makers to ensure that any surfaced problems were not left to linger for prolonged durations. Minutes of the meetings were also recorded and disseminated via email daily. 

An end-to-end encrypted instant messaging platform was also utilised in the daily dissemination of information and relaying of ground issues. In this way, tightly knitted communications and interventions were effected to facilitate optimal operational needs.

#### 2.3.3. Coordination

A system involving seamless coordination efforts both vertically within the hierarchical chain as well as horizontally across different members of the ground team was vital to effective daily operational processes and in reducing unnecessary wastage of “human manpower efforts”. The harnessing of computer algorithms in the daily operations in Big Box was evident from its usage in the bulk electronic pre-registration of the incoming patients in sync with the Ministry’s daily manifest, the generation and updating of each day’s clinical review tasks and handovers in real time, and checks over and above the automated cross-checking of fitness parameters in the decampment process of residents who were due for discharge. This hinged on the tight coordination between the central NUHS Academic Information Office (AIO) and the on-site CCF operations team who worked on the data input and processing. The usage of computer analytics and spreadsheets functioned as an objective means to decrease the propensity of human error factors as well as to reduce the reliance on human manpower for the processing and analysis of data. This translated to a better usage of the existing lean manpower and freed up the bandwidth of the clinical staff to deliver a better standard of care.

### 2.4. Operationalising the CCF: Readiness, Response and Recovery (The 3 R’s)

#### 2.4.1. Readiness

##### Setting up the CCF

Initial plans to set up a CCF were made in April 2020, and the mandate to manage the CCF was given to NUHS. In particular, the main task force comprising staff from JHC and Alexandra Hospital (AH) served as the core group who brainstormed and put together a multi-disciplinary team within a short time frame to join in the nation’s frontline battle against the pandemic. The core group consisted of medical, nursing and operations elements. The medical core group from JHC included experienced clinicians with relevant clinical and operational skills to manage the setting up of the healthcare facility. 

Standard operating protocols coupled with various workflows and guidelines were drafted and discussed at length to facilitate a smooth, safe and effective operation in a large community healthcare facility with inherent high-risk job scopes. 

To ensure the team was equipped and well poised for the tasks ahead, dedicated training sessions were carefully planned and tailored to the needs of each team member. Key members of the team were identified to be trained as super users, and expert resource personnel were to be team leads in subsequent training sessions and provide on the job support to the ground staff.

The training sessions included the following segments:

- Orientation
- Separate workflow briefings for medical, nursing and operations personnel
- Introduction and hands-on training for electronic medical records systems and analytics operations systems
- Basic cardiac life support (BCLS) recertification
- Personal protective equipment and N95 mask fitting training session
- Defibrillator and medical equipment training

Preparations together with extensive planning was accomplished within two months. A rigorous table-top exercise (TTX) laden with various clinical and operational scenarios was conducted two weeks prior to the actual opening with all major stakeholders to familiarise the entire team and to anticipate and troubleshoot potential contingencies. 

From the experience and knowledge gained from the TTX, further fine tuning and optimisation of existing protocols and workflows were made. These were again tested in a large-scale on-site full dress rehearsal (FDR) that was held one week prior to the actual site opening. 

Lessons and understanding gleaned from the FDR were used to further streamline the work processes. A detailed “Doctor’s Handbook for CCF/CRF@Big Box” was also published and issued to all doctors. This handbook contained all the salient information on the provision of care, workflows and processes. Finally, a licensing audit was conducted by the Ministry of Health to ensure the proper systems and process workflows were in place for the site opening.

##### Infrastructure and Manpower Structure: CCF/CRF@Big Box

The CCF@Big Box consisted of five halls spread across three levels. Level 1, with approximately 1400 beds, was managed by JHC. Level 3, with approximately 1000 beds, was managed by AH. At the time of writing, level 2, consisting of 600 beds, was not in operation.

**Figure 1:**
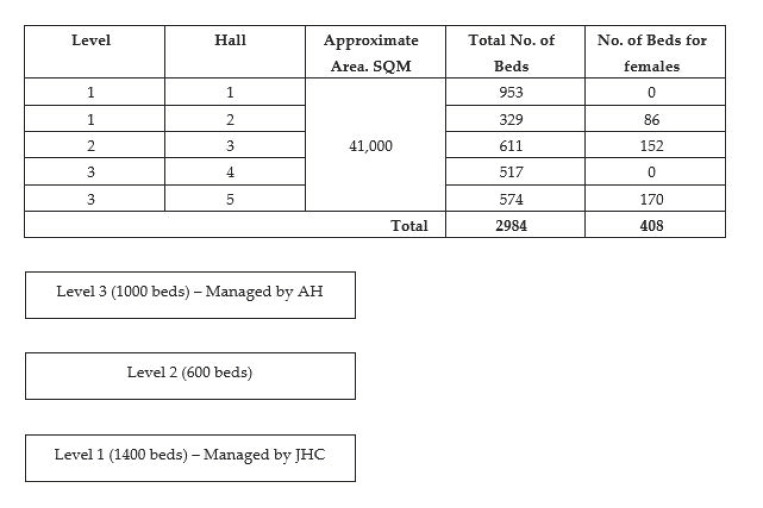
Infrastructure of the community care facility (CCF)@Big Box.

**Figure 2:**
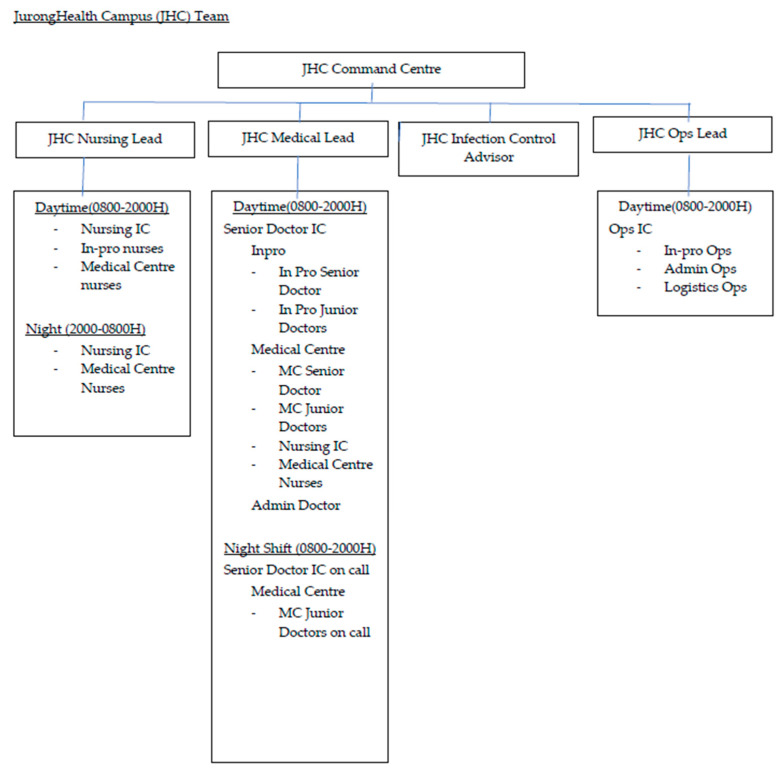
Structure of Jurong Health Campus (JHC) Team.

Figure 1 shows the layout of the CCF@Big Box. Figure 2 depicts the structure of the JHC Team.

##### Standard Operating Procedures

A common set of standard operating procedures (SOPs) was drafted and adopted by both JHC and AH teams. The SOPs served to describe the admission and discharge criteria, standards of care and escalation criteria for COIVD-19 patients admitted to the CCF/CRF@Big Box. The mode of care in the CCF/CRF provided essential primary healthcare services to a generally stable population with the aim of identifying early signs of complications arising from COVID-19 infection. 

**Figure 3:**
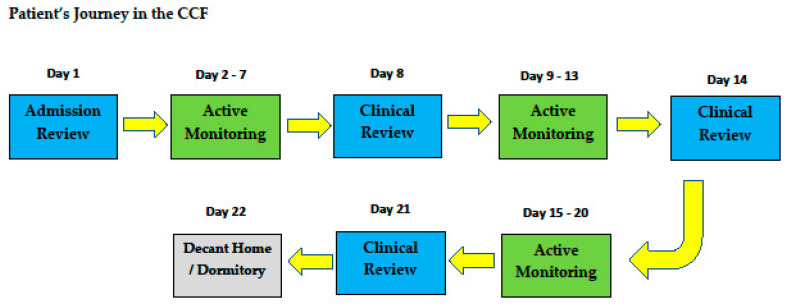
Patient’s journey in the CCF.

The schematic diagram depicting a typical resident’s journey to the CCF@Big Box is shown in Figure 3.

##### Workflows

**Figure 4:**
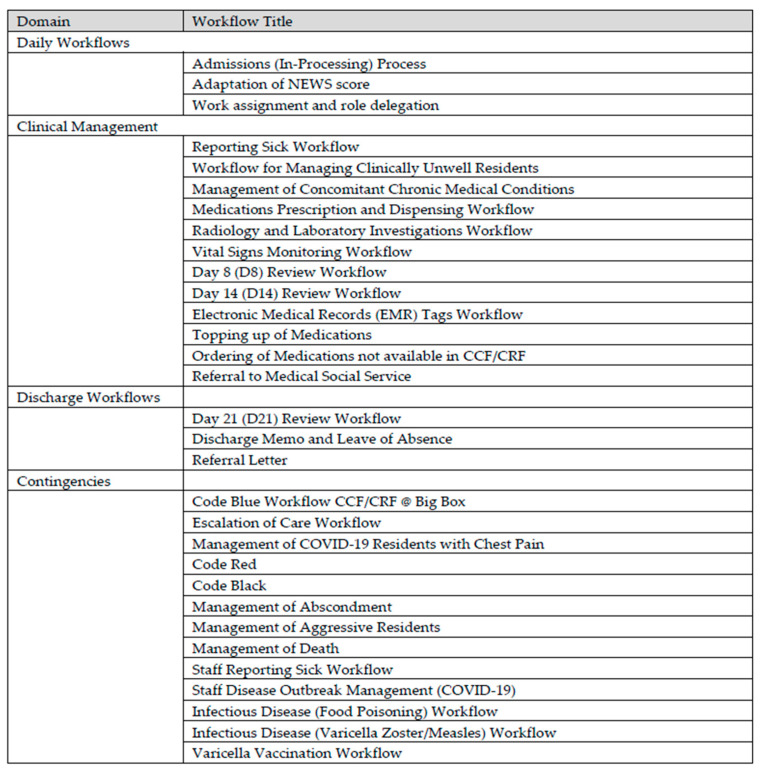
Workflows at the CCF/community recovery facility (CRF)@Big Box.

A repertoire of clinical and operational workflows was carefully drafted and collated. These workflows were designed to guide the ground team on the daily work processes and to maintain high care standards. As the management of COVID-19 is a rapidly evolving field, with newly updated knowledge and evidence made available every day, the workflows were progressively and continually updated and made relevant by the team on a regular basis. Inputs and advice from various domain experts were sought and adapted into the workflow considerations. A table depicting some of the important workflows is illustrated in Figure 4.

##### Team Structure

The team was recruited and put together within short notice, at a time when healthcare resources were stretched thin due to the ongoing pandemic. 

The clinical, operations leads and core groups were assembled from various department leads in the Jurong Health Campus, which is under the National University Healthcare System Cluster in Singapore. Of note, many of the volunteers from Allied Health and healthcare administration departments stepped up to contribute their time and service to this national need. Special attention was given in the selection considerations to ensure minimal disruptions and gaps of service in the organizational structures from which these personnel were seconded. A large proportion of the clinical staff (e.g., doctors and nurses) was recruited from various sectors, including private clinics and institutions as well as the locum pool. Interestingly, the different backgrounds of the team members varied widely. For instance, among the doctors, there were private practice specialists, public institution specialists, community hospital family physicians, aesthetic medicine clinicians, private general practitioners, and part-time hospital and clinic doctors. Among the nurses, there were senior advanced practice nurses, nurse clinicians, nurse educators, staff nurses, assistant nurses as well clinic managers from both the public and private healthcare sectors. In the operations team, there were individuals from operations; Allied Health professionals such as physiotherapists, speech therapists and podiatrists; and administrative and managerial staff from various hospital support functions such as human resources and clinical education offices.

Though the team members were from various walks of life, professions, ages and life experiences, their cohesiveness, team spirit, commitment and synergy cannot be overstated. Regardless of seniority, hierarchical rank or experience, the team huddled together to serve a common purpose: that of putting in their best as a frontline pandemic workers, undeterred by long working hours involving 12 hour shifts, prolonged periods of work while being in full personal protective equipment (PPE), and the risks of dealing with an infectious, yet invisible enemy—SARs-Cov-2. 

It was heartening to see many senior team members taking great care to lead by example and look after their junior colleagues, with a common daily objective: that is, to complete the call of duty to care for the COVID-19 residents and to return them home safely to their loved ones. Facing hundreds of admissions and sick consults daily, the team forged ahead as a unit to complete the task at hand, looking after one another in the process and making sure everyone had their regular mask breaks and meal times.

#### 2.4.2. Response

The CCF/CRF@Big Box was officially open for operations on 4 July 2020.

##### Shift Parade Briefings

Each shift commenced with team briefing sessions from the medical, nursing and operations leads on duty. Attendance and the well-being of each team member was assessed prior to the start of each working shift. The projected workload of the shift was shared, and schedules on the rotation of manpower into the red zones were discussed and strategized. Important announcements and updates from the command team and previous days’ debriefing meetings were disseminated during this session. Time was also given to solicit feedback on any issues encountered by the ground team. Such feedback would be dealt with immediately by the team leads, who would relay specific issues at the command team debriefs at the end of each day.

##### Operational Strategies

In view that the workflows and processes were new and unfamiliar to most of the team members who were assembled at short notice from varied backgrounds, various operational and training strategies were utilised to bridge the gaps in knowledge and smoothen the day-to-day work processes. 

##### Usage of Flowcharts and Key Visual Cues Charts

**Figure 5:**
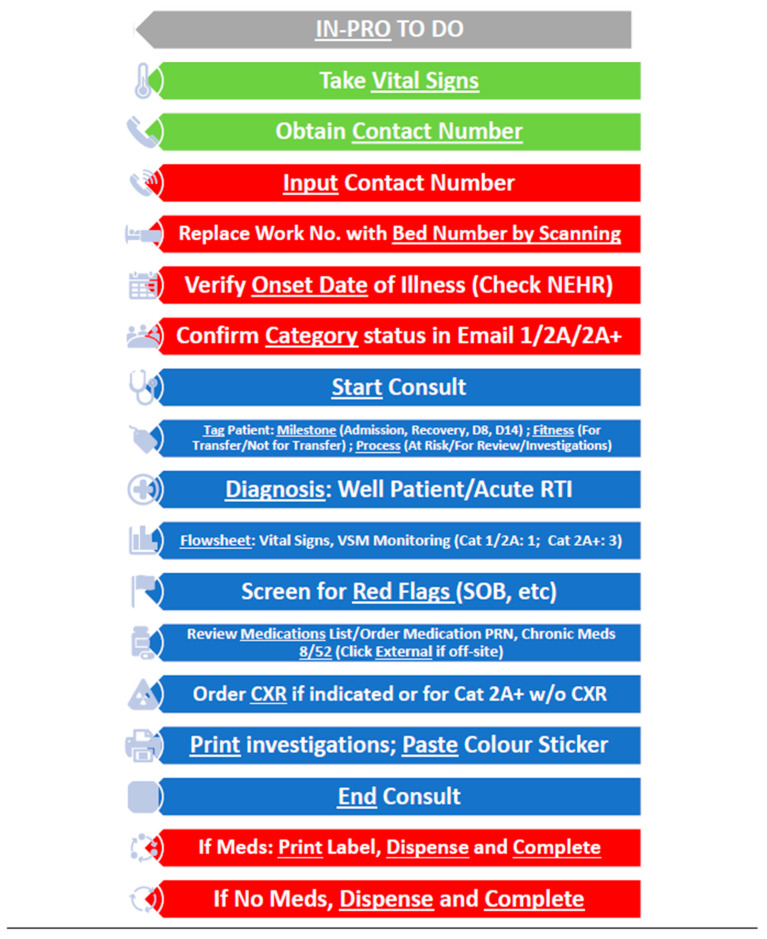
Visual cue charts at In-Pro. Legend: green: nursing tasks; red: clinic management system tasks; blue: electronic medical record tasks.

**Figure 6:**
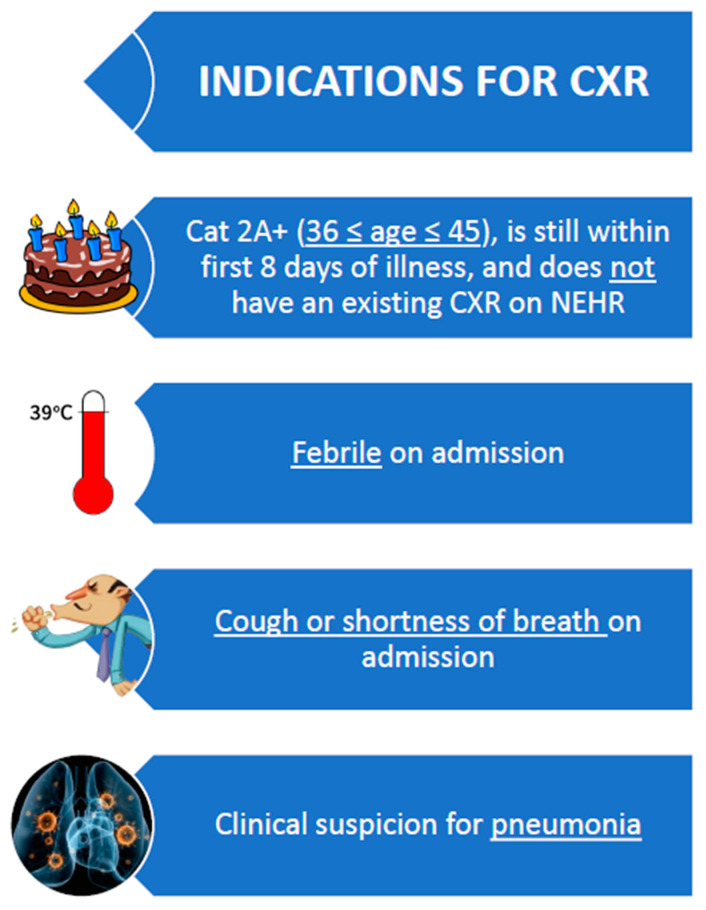
Visual cue charts at consultation stations: indications for chest radiograph.

Specially designed flow charts summarising the common workflow processes (e.g., In-processing, report sick, Day 8/14/21 reviews, etc.) were printed and strategically placed at each consultation station to function as both visual cues for the users as well as quick reference materials that came in handy in daily work operations. Important disease categorisation charts, dichotomous designed charts helping clinicians in their determination of disease onset dates based on symptoms, swab and serology results, and the available pharmacopoeia at the facility were similarly made accessible within the consultation stations. Examples of the visual cue charts are found in Figure 5 and Figure 6. 

##### Pre-Packed Medications

Medications for common ailments were prepacked into specific categories and supplied at each consultation station. This allowed swift ordering and dispensing of such medications at the point of consultation, reducing the overall wait times for each resident, negating the need for a separate dispensary stations involving commonly used medications, and minimising the touchpoints of different staff with residents who were being treated for COVID-19.

##### Electronic Repository Database

There was a need for an efficient database for the tracking of clinical follow-ups, handovers, results and communications across team members in red and green zones, as well as the administrative team. Of importance was avoiding the usage of hard copies as well as reliance on any manual tracking in order to adhere to strict infection control measures and to minimise potential gaps in care delivery through human error. 

No paper records were taken out of the red zone. Tracking of radiology forms was also done virtually. As a result, the risk of fomite contamination was kept to a minimum. 

In addition to the tracking of clinical and results follow-up, the database also had inbuilt algorithms to cross-check for errors and missing data. This formed another level of safeguard to ensure that a patient’s continuity of care was not compromised. 

##### Staff Safety, Welfare and Morale

Safety and Security

Staff safety was undoubtedly of utmost importance during the course of planning and actual work. Strict infection control practices, albeit cumbersome and ergonomically uncomfortable, were strictly enforced, complied with and audited. Attentive logistic support ensured adequate supplies of PPE as well as clinical equipment, with built-in redundancy to cope with any unexpected surge. Twice daily temperature screening and reporting of all staff, regardless of their working status, with the compulsory usage of safe-entry (a national initiative to facilitate contact tracing) were in force. Clear demarcation of the red and green zones within the compound was enforced and staffed by the on-site auxiliary police security team, which not only kept safety and infection control measures in check but also ensured the physical safety of all staff during work.

Welfare, Morale and Staff Support

Staff welfare and their psychological well-being was never taken for granted. A multinational, multicentre study on the psychological outcomes and associated physical symptoms of healthcare workers during the COVID-19 pandemic demonstrated that adverse psychological outcomes and physical symptoms were significantly associated with the pandemic [11].

Other studies have also shown a similar trend, raising awareness of the negative psychological outcomes and the need to look after the psychological health of healthcare workers during the COVID-19 pandemic [12,13,14]. 

Some studies have even shown a propensity to develop de novo headaches or exacerbation of pre-existing headache disorders with the prolonged usage of PPE among frontline healthcare staff [15].

Thus, a serious stance and emphasis on looking after and safeguarding the welfare, morale and psychological well-being of all staff was in place. Regular engagement sessions between the team and senior management as well as walk-abouts from senior management and ministerial staff were evident to listen to feedback from the ground staff as well as to encourage and recognise the contributions and sacrifices made by the team. Frequent snacks and treats were also delivered from the central command team or sponsored by the senior staff on duty to reward and perk up the team in their daily course of work. Daily debrief huddles and end-of-day reviews together with the command team in attendance served as a platform not only to relay information and tasks, but also to solicit feedback from the ground on issues and difficulties faced so that timely interventions and assistance could be rendered to mitigate the ground stressors. A common piece of feedback received was that the team felt heard and supported throughout their course of work at the COVID-19 facility. Senior team members were also trained and empowered to look out for signs of stressors among team members who might not be coping well. An encouraging observation was the frequent sightings of and comments about senior team members going beyond their scope of duty to render assistance to juniors in their daily work as well as making sure that no team member was left behind alone to cope with remaining day’s work.

Interestingly, similar studies have been conducted and have shown that many of the above-mentioned morale-boosting measures and recognition of efforts led to a morale boost during the pandemic [16].

#### 2.4.3. Recovery

The battleplan against COVID-19 was forecasted to be long-term. There was a pressing need for members of the founding team to return to their original institutional duties to attend to their patients once the foundation and operations of the CCF had been set up and running smoothly. 

As such, within the first month of actual CCF operations, a private healthcare group entity was engaged to prepare and take over the site operations. Adequate time, training and orientation sessions were arranged so that they could understudy and shadow the existing team in carrying out their various duties and responsibilities. 

On 3 August 2020, JHC handed over the level 1 operations to the private sector team; this was followed by AH handing over the level 3 operations a month later.

## 3. Results

### 3.1. Attendance

Over the one month of CCF operations by JHC, there was a total of 1341 admissions. In addition, we also attended to 788 ad hoc physical clinical consultations when medical complaints arose during the course of admission, resulting to a total of 2129 clinical encounters (Table 1).

### 3.2. Demographics

Of the patients, 71.8% (963 of 1341) were between 17–35 years of age, 24.8% (333 of 1341) were between the age of 36–45 years and the rest were above 45 years old. All of the patients were male. 51.6% (692 of 1341) of the patients were from India and 34.4% (462 of 1341) from Bangladesh (Table 2).

The majority of the patients (93.4%, 1252 of 1341) were between day 2 and day 6 of illness upon CCF admission. The rest of the patients (6.6%) were at day 7 of illness or beyond (Table 3).

### 3.3. Outcome of Admissions

Overall, there was a good outcome for patients who were managed at the CCF. Of note, the vast majority remained well and progressed uneventfully, with only 2.3% (31 of 1341) of patients requiring transfer back to restructured hospitals for further management. Out of these 31 patients, 22.6% (7 of 31) of patients returned to the CCF after hospital consultation. Within the 31 patients who were referred to restructured hospitals, 9.7% (3 of 31 patients) were sent via emergency ambulance, while 90.3% (28 of 31 patients) were sent via non-emergency ambulances. There were no cases of mortality or cases that presented with significant morbidity. 

Of the patients, 259 (19.3%) were discharged by 2 Aug 2020. At the point of discharge, two patients (0.8%) were discharged prior to day 14 of illness, five patients (1.9%) were discharged on day 14, 67 patients (25.9%) were discharged between day 15 and day 21 of illness, and the remaining 185 patients (71.4%) were discharged after day 21 of illness (Table 4).

### 3.4. Resources Used

A total of 362 radiological procedures and six laboratory tests were performed. 

With regard to the need for pharmacy support, a total of 113 external prescriptions (medications that were not available on site) were issued, including 54 external chronic medication prescriptions belonging to 40 individual patients.

### 3.5. Engagement Sessions for Psychological Support

A total of five resident engagement sessions involving 200 residents were conducted. Three of the sessions involving 120 residents were conducted in Tamil, while two of the sessions involving 80 residents were conducted in Bengali. 

In addition, there were two exercise sessions involving 80 residents as well as one session of art therapy with 40 participants. 

Nine residents required Healthserve referral for further psychological support.

## 4. Challenges and Lessons Learned

### 4.1. Thresholds and Standards Setting Across Diverse Backgrounds and Experiences

One of the imminent challenges faced by the medical team was the difficulty in calibrating and standardising the threshold for escalation of care as well as the standards of chronic disease management and primary care condition management. As previously mentioned, our professionals were sourced and assembled from varied and diverse backgrounds and experiences. Thus, it was not easy to get everyone to agree on or to be comfortable with the management of conditions involving varying levels of severity and complexity within a limited and short duration of on-the-job training. Yet, despite the inherent challenges faced, it was heartening to witness moments of humbleness and willingness to help one another to overcome challenges. For instance, it was common to see a family physician or general practitioner sharing information with their surgically trained or more junior colleagues on the management of chronic diseases and common primary care complaints. Similarly, it was common to see an emergency medicine specialist or institutional specialist stepping up to assist and coordinate the management of medical emergencies and complex conditions with colleagues who might not be familiar or confident with such processes. 

### 4.2. Manpower Constraints and Workload Mismatch

It is not surprising to note that, because the team was assembled within a very short time in the context of a pre-existing stretched and limited resource pool, there was little redundancy available to cross cover or replace the duties of other colleagues in the event of sick leave or urgent family matters. However, the impact of last-minute manpower contingencies was mitigated by the following measures. Fellow colleagues went to great extents to accommodate one another’s availabilities and swapped their shifts or even did additional shifts when recalled, minimising disruptions to the ground manpower. Team members readily agreed to cover one another when the need arose or when contingencies occurred. 

On many days, it was not uncommon to see workload mismatch between the different teams. As the number of admissions varied and peaked on different days, which was impossible to predict from a rostering perspective, there were days when an extreme surge in admission numbers overwhelmed the team handling the admissions. However, these potential pain points always seemed to be averted when spontaneous volunteering and help from other teams as well as other institutions present occurred, even without the need for interventions from the command centre. This frequent sight of unity amid pandemic adversity was certainly commendable.

### 4.3. Addressing the Invisible Enemy—Psychological Well-Being

It was imperative to look after the psychological well-being of our staff. Given the many uncertainties that were associated with COVID-19, it is understandable that many staff experienced stress and were concerned about risk of infection as well as the well-being of their loved ones when they returned home from work. To address this concern, supplies of all personal protective equipment were made readily available among the entire team. Dedicated ante-rooms with adherence to recommended pressure settings as per infection control protocols were built and maintained throughout the duration of CCF operations. Ample shower facilities as well as surgical scrub suits with laundry service were provided for staff usage. For selected staff who were staying with vulnerable family members, special accommodations were arranged. 

Apart from addressing the psychological concerns of our staff, it might have been easy to overlook that COVID-19 stricken residents were probably facing a greater toll on their mental well-being, with much anxiety and uncertainty. 

Indeed, a significant proportion of the admitted residents were foreign workers who may not have been well-educated and may have had significant family commitments to fulfil financially. It was a common sight to see patients who were not even aware that they had been tested positive for COVID-19, even at the point of medical triage at admission. Language barriers and knowledge gaps aside, it was not difficult to fathom that these patients were stricken with much fear, uncertainty and concern about their prognosis, progress and even their job stability. 

The team was able to take care of the medical aspects of their recovery and provide optimal care. However, the more challenging task was to also address psychological issues and concerns, both adequately and comprehensively. The team recognised the importance of addressing this aspect of the residents’ care. Extensive engagements with medical social services (MSS) as well as Healthserve, a non-government organisation that is active in looking after the well-being and rights of foreign workers in the Singapore community, led to the culmination of formal workflows where active screening for relevant anxiety and other psychological symptoms were performed at the point of admission for each resident as well as during opportunistic ad hoc consults at the medical centre. Should the screening outcome be positive, the resident was offered to be linked up with MSS or Healthserve as well as given a follow-up assessment to determine if further treatment or intervention was required. During the admission process and consultation, efforts were also made to explain the reason for the encounter and the projected course of illness to empower each resident in addition to allaying their anxiety and fears. 

Furthermore, regular engagement sessions with the residents conducted in their native language were organised to address any of their concerns as well as to identify potential stressors needing intervention and follow-up. 

Efforts were also made to engage the residents through provision of their native treats, leisure boardgames as well as conducting mass exercise sessions. These soft touches were important and relevant elements of psychological support for the residents and a reminder for us that we were there to cure sometimes, treat often and comfort always.

## 5. Discussion

### 5.1. Experiences from the CCF/CRF@Big Box

From our experience at the CCF/CRF@Big Box, the importance of having a blueprint of public health emergency preparedness, which can be operationalised upon short notice, cannot be over-emphasised. The operational structure, workflows, protocols and tested strategies gleaned from this experience can be systematically chronicled and templated to serve as ready trialed and tested standard operating procedures that can be adapted for quick implementation to address potential future public health disease outbreaks or pandemics. During the initial planning phase of our operations, much effort and manpower hours were put into drafting of the protocols and workflows from scratch. Even after the subsequent implementation, it took several rounds of on-the-job revisions to fine-tune the processes and make them relevant and efficient for the ground team. The chronicled experiences together with archived work processes will result in substantially reduced efforts and delays in adapting similar operations in the future. 

It is also evident from the CCF@Big Box that we can think “out of the box" in adapting ready-constructed commercial buildings into “field hospitals”. The purpose is to right-site the affected population who requires minimal medical attention to community facilities and thus free up the tertiary hospitals to handle patients who need advanced care and monitoring. Healthcare resources as well as facilities are valuable, yet expensive to maintain. Through this experience, it was successfully demonstrated that it is possible to re-configure appropriately selected existing infrastructure, with minimal disruptions, into temporary healthcare facilities to meet the needs of public health emergencies, thus negating the need for massive hospitals to be specially built to cope with such a surge in needs at short notice.

It also demonstrated the importance of harnessing the strengths of the various healthcare stakeholders and resources to complement public health needs during unexpected emergencies. For instance, in the initial teething phase of gathering the core team in planning and operationalising a huge healthcare facility, it was apt to tap into the existing public healthcare front liners who were familiar with the current public healthcare policies and requirements, empowering them to lay a strong foundational framework prior to the gradual tapping of the private sector partners in taking over and continuing the existing stable operations. Such a strategy allowed the utilization of already available resources with the relevant expertise and knowledge to be assembled hierarchically into functionally efficient pioneer teams. This allowed the gradual recruitment of resources from the private sector to take over the transition phase with firmer foundations and allowed the pioneer group to return to their primary duties or to lead a second operation using a similar strategy. 

Through our experience in this CCF operation, we also learned to appreciate the importance of looking after the emotional well-being of staff and patients alike. The threat of facing many unknown risks can sometimes have a more debilitating effect on the morale and resilience than the disease itself. The necessity to have self-care and engagement touchpoints built within the system to address these needs must be emphasised. 

It is noteworthy that over the course of our operations in the Big Box CCF, none of our staff was infected by the COVID-19 virus. This is the testament to the infection control measures put in place, the surveillance system to ensure staff compliance with these measures and also the maturity and understanding of our staff of the importance of these measures. We witnessed reports of healthcare workers coming down with COVID-19 in the course of their work for various reasons, and thus the importance of staff safety was drummed into all our teams [16,17,18]. There were also constant reminders and surveillance to ensure compliance. 

While we established the workflows and tested them prior to launching our operations, we realised quickly that the hot battle with the virus was a rapidly evolving one. This was evident as new knowledge and new operational needs evolved as we progressed. Furthermore, we also had to balance the operational needs of our partners and the needs of our base hospital. There was thus a need to be responsive and react decisively when the need arose.

While the focus was initially on the disease itself, it was evident to us that taking care of the social and psychological needs of our residents was equally important. Bearing in mind that these residents were mostly well physically, we had to ensure that their social and psychological needs were catered to as well. There were efforts to engage them in their native languages at organised focused groups; also, group activities like exercise and art therapy sessions were used to keep them engaged. This allowed us to ensure the overall well-being of our residents. 

### 5.2. Comparison with Other Global COVID-19 Mass Isolation Facilities

#### 5.2.1. Repurposed Large-Scale Facility

The operational concept in our CCF was similar to other CCFs set up within Singapore, such as the CCFs located at Singapore Expo by Woodlands Health Campus as well as Singhealth [19,20]. All three CCFs converted existing large-scale facilities into mass isolation facilities. The pre-existing infrastructure coupled with existing electrical and water supply networks allowed seamless and prompt conversion with minimal construction efforts and planning. This allowed CCFs to be operationalized within a short time frame, which is vital in the containment of infectious patients during a pandemic.

Similarly, in Korea, community treatment centers (CTCs) were repurposed from existing buildings. Gyeongbuk-Daegu 7 CTC was one of the largest CTCs set up in Korea to deal with the COVID-19 pandemic; it was repurposed from a previous dormitory [9,21]. In Wuhan, China, similar concepts involving Fangcang shelter hospitals, with a 1600 bed capacity, were refurbished into temporary healthcare sites from existing exhibition centres and stadiums [7,8]. In addition, Wuhan KeTing Medical shelter hospital was built from the previous Wuhan Culture and Art Center, with a bed capacity of 1461 [22].

Modelled after Fangcang shelter hospitals, an alternative hospital site (AHS) was built from an existing convention center in Rhode Island, US, within a month’s duration. This repurposed 600 treatment beds for the care of patients requiring isolation [10]. 

#### 5.2.2. Patient Profile

Our CCF emphasised functioning as a mass isolation facility to provide basic medical care and surveillance for COVID-19 patients who were largely well and ambulant; reliance on patient ownership and empowerment through basic health education and self-directed vital sign measurements; looking after the psychological well-being of the patients. A similar patient profile is seen across the CCFs in Singapore [19,20]. 

The CTC in Korea focused mainly on the isolation and active surveillance of patients with mild symptoms of COVID-19 who did not require advanced medical resources. This is similar to the patient pool in our CCF [9,21]. In the shelter hospitals set up in Wuhan, staff attended to patients presenting with mild to moderate symptoms of the disease.

In the AHS in Rhode Island, patient inclusion criteria were restricted to those 18 to 65 years of age who were largely stable in their disease progression [10]. 

#### 5.2.3. Process

The CCF@Big Box utilised an adapted electronic medical records system that was linked to a shared nationwide healthcare database to streamline continuity of care and minimize cap gaps when patients travelled to and from our CCF. This is similar to what was adopted at the CCFs by Woodlands Health Campus and Singhealth. In all these CCFs, it was the norm for patients to have their vital signs self-taken at designated vital sign stations, which were routed electronically to the central monitoring system [19,20]. 

In the Korean CTCs, patients utilised a special mobile application as well as telephone communications to report their vital signs. An electronic medical information system was also used [9,21]. 

#### 5.2.4. Outcome

During the month’s duration of operation at the CCF@Big Box, only 2.3% (31 of 1341) of patients required transfers back to restructured hospitals for further management. This was comparable to the slightly higher rate of 3.6% (n = 136) of patients requiring transfer back to acute hospitals seen at the CCF@Expo managed by Singhealth [20]. The CCF managed by Woodlands Health Campus reported a transfer-out rate of 0.37%; however, there was no available detail regarding whether this was inclusive of the patients in CCF@Expo managed by Singhealth [19]. Nonetheless, the similarity of having a low rate of patients requiring transfers to restructured hospitals was evident among the CCFs, reflecting the success in the right-siting of ambulatory care to reduce burden and workload in acute hospitals [19,20].

In the CTC in Korea, 2.3% of the patients required transfer back to hospitals [9]. This was similar to the transfer-out rate at the CCF@Big Box. Interestingly, the Fangcang shelter hospitals in Wuhan reported a 13% rate of admitted patients who were transferred to a higher level hospital. This appears to be much higher than what was seen in similar facilities in Singapore and Korea. However, there is inadequate detail provided that might account for this difference. One possible reason might be the fact that Fangcang shelter hospitals were among the earliest to be set up; the CCFs and CTCs that subsequently began operation were probably able to model after the successes of the Fangcang pioneers and build upon their shared experience in averting undesired outcomes.

#### 5.2.5. Uniqueness and Advantages of the CCF@Big Box

The CCF@Big Box was fortunate to record no patients with fatalities. Overall, in the CCF@Singapore Expo, one patient passed away two weeks after discharge due to pulmonary thromboembolism post-COVID-19 [20]. The zero mortality rate in CCF@Big Box and remarkably low mortality rate in the CCF@Singapore Expo are a testament to the satisfactory level of medical care that was rendered to the large number of patients within the mass isolation facilities in Singapore. It also reflects the appropriateness in the national strategy in conceptualising the CCF operations in mitigating pandemic needs involving disease of reasonably lower fatality rates, and alleviating hospital congestion and empowering acute hospitals to continue providing their crucial healthcare services without being overwhelmed.

The CCF@Big Box was unique in that it involved the transformation of a pre-existing large commercial building, which was already left unoccupied, with an almost complete infrastructure that only required minor modifications to be operationalised as a healthcare facility within a very short time frame. The other Singapore CCFs, Korean CTCs, Wuhan’s Fangcang shelter hospitals and Rhode Island’s AHS were all converted from facilities that were in current usage and thus probably incurred more logistical and construction planning in converting them.

To the best of our knowledge at the time of writing, the CCF@Big Box was the only repurposed large-scale COVID-19 healthcare community facility that was co-located with an existing tertiary hospital. Ng Teng Fong General Hospital is one of the tertiary hospitals in Singapore. It is located right opposite the CCF@Big Box, with a connecting overhead bridge. This presented substantial logistical and manpower advantages in the transportation of supplies and conveyance of patients bi-directionally, from the acute hospital to the CCF as well as escalation of care from the CCF back to the acute hospital. Quality and turnaround time of laboratory specimens could also be better maintained as the transport route from the CCF to the hospital laboratory took less than a minute by road. The same could be said of dispensing of non-formulary medications from the hospital pharmacy to the CCF. The other CCF@ Singapore Expo was located kilometers away from the nearest tertiary hospital.

The CCF@Big Box also placed huge emphasis on staff communications. The clockwork daily shift parade team briefings as well as the command team meetings were exceptionally crucial in dissemination of important information and rapidly changing protocols to the entire team. The daily command team meetings also served as continual direct feedback of the challenges encountered by the ground team to the management team, who was able to rapidly clear obstacles and provide the necessary resources, negating the traditional inefficient channels of multiple-level reporting. 

The unique usage of visual cue workflows and flowcharts in the CCF@Big Box enabled less reliance on providers’ rote memory, minimized errors and led to better efficacy of the novel operational systems in the quest to deliver a better quality of care. 

## 6. Conclusions

The Big Box CCF saw and managed 1341 patients within the first month of operation. The majority of the patients remained well, with no deaths or major complications encountered. The attention to preparation, organisation and day-to-day processes resulted in the smooth flow within the CCF. The consideration of psychological concerns and effects on patients and the healthcare team through active preventive measure was vital. 

The battle against SARS in 2003 set the groundwork for many countries like Singapore to prepare for the possibilities of pandemics [3,23]. The experiences from the ongoing yet likely long-term war against COVID-19 will be invaluable and imperative to mould our future readiness and response to a similar resurgence of the pandemic or even with other potential infectious agents that threaten the security of our people. The authors of this article hope that we can share both the positive and negative learning experiences across global COVID-19 teams so that we can emerge stronger and wiser in our future preparedness. 

Unity is often best displayed when facing adversity.

## Figures and Tables

**Table 1:** Number of patients seen at the CCF/CRF@Big Box from 4 July 2020 to 2 August 2020 based on type of medical consultation.

Type of Consultation	Number of Patients	Percentage of Total Consults
Admission review	1341	63.0%
Day 8 physical review	4	0.2%
Day 14 physical review	11	0.5%
Ad-hoc physical review	11	0.5%
Scheduled appointments	129	6.1%
Walk-ins	633	29.7%
**Total**	**2129**	**100%**

**Table 2:** Demographics of patients admitted to the CCF/CRF@Big Box from 4 July 2020 to 2 August 2020 (sorted by nationality).

Nationality	Number of Patients	Percentage of Total
Bangladesh	462	34.5%
Burma	11	0.8%
China	28	2.1%
India	692	51.6%
Indonesia	1	0.1%
Malaysia	3	0.2%
Myanmar	29	2.2%
Philippines	3	0.2%
Sri Lanka	1	0.1%
Thailand	6	0.4%
Unknown	105	7.8%
**Total**	**1341**	**100%**

**Table 3:** Number of patients admitted to the CCF/CRF@Big Box from 4 July 2020 to 2 Aug 2020 based on their day of illness upon arrival to the CCF.

Day of Illness upon Arrival at CCF	Number of Patients	Percentage of Total
2	93	6.9%
3	245	18.3%
4	261	19.5%
5	365	27.2%
6	288	21.5%
7 or more	89	6.6%
**Total**	**1341**	**100%**

**Table 4:** Number of patients discharged from the CCF/CRF@Big Box from 4 July 2020 to 2 Aug 2020 based on their day of illness on discharge from the CCF.

Day of Illness upon Discharge from CCF	Number of Patients	Percentage of Total
<14 days	2	0.8%
14 days	5	1.9%
15–21 days	67	25.9%
>21 days	185	71.4%
**Total**	**259**	**100%**

## Data Availability

Data sharing not applicable. No new data were created or analyzed. Data sharing is not applicable to this article.

## References

[1] Cucinotta D., Vanelli M. (2020). WHO Declares COVID-19 a Pandemic. Acta Biomed..

[2] Chauhan S. (2020). Comprehensive review of coronavirus disease 2019 (COVID-19). Biomed. J..

[3] Lin R.J., Lee T.H., Lye D.C. (2020). From SARS to COVID-19: The Singapore journey. Med. J. Aust..

[4] Remarks by Minister Lawrence Wong, Co-chair of the Multi-Ministry Taskforce on COVID-19, at Press Conference on COVID-19 at National Press Centre on 5 April 2020. https://www.sgpc.gov.sg/sgpcmedia/media_releases/mnd/speech/S-20200405-1/attachment/Remarks%20by%20Minister%20Lawrence%20Wong%20at%205%20Apr%20Press%20Conference%20on%20COVID-19.pdf.

[5] 9 in 10 Coronavirus Patients in Singapore Housed in Community Facilities, The Straits Times, Published 25 April 2020. https://www.straitstimes.com/singapore/9-in-10-coronavirus-patients-housed-in-isolation-facilities.

[6] Coronavirus: Singapore Expo to House First Batch of Mild Cases Tomorrow to Free up Hospitals, The Straits Times, Published 9 April 2020. https://www.straitstimes.com/singapore/health/spore-expo-to-house-first-batch-of-mild-cases-tomorrow-to-free-up-hospitals.

[7] Chen S., Zhang Z., Yang J., Wang J., Zhai X., Bärnighausen T., Wang C. (2020). Fangcang shelter hospitals: A novel concept for responding to public health emergencies. Lancet.

[8] Fang D., Pan S., Li Z., Yuan T., Jiang B., Gan D., Sheng B., Han J., Wang T., Liu Z. (2020). Large-scale public venues as medical emergency sites in disasters: Lessons from COVID-19 and the use of Fangcang shelter hospitals in Wuhan, China. BMJ Glob. Health.

[9] Park P.G., Kim C.H., Heo Y., Kim T.S., Park C.W., Kim C.-H. (2020). Out-of-Hospital Cohort Treatment of Coronavirus Disease 2019 Patients with Mild Symptoms in Korea: An Experience from a Single Community Treatment Center. J. Korean Med. Sci..

[10] Naganathan S., Meehan-Coussee K., Scott Pasichow M.D., Rybasack-Smith H., Binder W., Francesca Beaudoin M.D., Musits A.N., Sutton E., Petrone G., Levine A.C. (2020). From Concerts to COVID: Transforming the RI Convention Center into an Alternate Hospital Site in under a Month. Rhode Isl. Med. J..

[11] Chew N.W., Lee G.K., Tan B.Y., Jing M., Goh Y., Ngiam N.J., Yeo L.L., Ahmad A., Khan F.A., Shanmugam G.N. (2020). A multinational, multicentre study on the psychological outcomes and associated physical symptoms amongst healthcare workers during COVID-19 outbreak. Brain Behav. Immun..

[12] Wang C., Pan R., Wan X., Tan Y., Xu L., Ho C., Ho R. (2020). Immediate Psychological Responses and Associated Factors during the Initial Stage of the 2019 Coronavirus Disease (COVID-19) Epidemic among the General Population in China. Int. J. Environ. Res. Public Health.

[13] Li Z., Ge J., Yang M., Feng J., Qiao M., Jiang R., Bi J., Zhan G., Xu X., Wang L. (2020). Vicarious traumatization in the general public, members, and non-members of medical teams aiding in COVID-19 control. Brain Behav. Immun..

[14] Kang L., Ma S., Chen M., Yang J., Wang Y., Li R., Yao L., Bai H., Cai Z., Yang B.X. (2020). Impact on mental health and perceptions of psychological care among medical and nursing staff in Wuhan during the 2019 novel coronavirus disease outbreak: A cross-sectional study. Brain Behav. Immun..

[15] Jy Ong J., Bharatendu C., Goh Y., Zy Tang J., Wx Sooi K., Lin Tan Y., Tan B., Teoh H.L., Ting Ong S., Allen D.M. (2020). Headaches associated with personal protective equipment-a cross-sectional study amongst frontline healthcare workers during COVID-19 (HAPPE Study). Headache.

[16] Barranco R., Ventura F. (2020). Covid-19 and infection in health-care workers: An emerging problem. Medico-Leg. J..

[17] Razzak J., Bhatti J.A., Tahir M.R., Pasha-Razzak O. (2020). Initial estimates of COVID-19 infections in hospital workers in the United States during the first wave of pandemic. PLoS ONE.

[18] Lancet T. (2020). COVID-19: Protecting health-care workers. Lancet.

[19] Goei A., Tiruchittampalam M. (2020). Community Care Facility—A Novel Concept to Deal With the COVID-19 Pandemic: A Singaporean Institutions Experience. J. Public Health Manag. Pract..

[20] Chia M.L., Chau D.H.H., Lim K.S., Liu C.W., Tan H.K., Tan Y.R. (2020). Managing COVID-19 in a Novel, Rapidly Deployable Community Isolation Quarantine Facility. Ann. Intern. Med..

[21] Her M. (2020). Repurposing and reshaping of hospitals during the COVID-19 outbreak in South Korea. One Health.

[22] Yu H.P., Ma L.L., Hung Y.Y., Wang X.B., Peng Y.Q., Zhuang H.R. (2020). Application of ‘mobile hospital’against 2019-nCoV in China. Epidemiol. Infect..

[23] Goh S.S., Chia M.Y. (2020). Anxiety and Morale in Front-Line Healthcare Workers during the Coronavirus Disease 2019 (COVID-19) Outbreak at the National Screening Centre in Singapore. Ann. Acad. Med. Singap..

